# NMR metabolomics highlights sphingosine kinase‐1 as a new molecular switch in the orchestration of aberrant metabolic phenotype in cancer cells

**DOI:** 10.1002/1878-0261.12048

**Published:** 2017-03-30

**Authors:** Caterina Bernacchioni, Veronica Ghini, Francesca Cencetti, Lukasz Japtok, Chiara Donati, Paola Bruni, Paola Turano

**Affiliations:** ^1^ Department of Biomedical, Clinical and Experimental Sciences University of Florence Italy; ^2^ CERM and Department of Chemistry University of Florence Italy; ^3^ Department of Toxicology Faculty of Mathematics and Natural Science Institute of Nutritional Science University of Potsdam Germany

**Keywords:** NMR‐based metabolomics, ovarian cancer, sphingosine kinase‐1, Warburg effect

## Abstract

Strong experimental evidence in animal and cellular models supports a pivotal role of sphingosine kinase‐1 (SK1) in oncogenesis. In many human cancers, SK1 levels are upregulated and these increases are linked to poor prognosis in patients. Here, by employing untargeted NMR‐based metabolomic profiling combined with functional validations, we report the crucial role of SK1 in the metabolic shift known as the Warburg effect in A2780 ovarian cancer cells. Indeed, expression of SK1 induced a high glycolytic rate, characterized by increased levels of lactate along with increased expression of the proton/monocarboxylate symporter MCT1, and decreased oxidative metabolism, associated with the accumulation of intermediates of the tricarboxylic acid cycle and reduction in CO_2_ production. Additionally, SK1‐expressing cells displayed a significant increase in glucose uptake paralleled by GLUT3 transporter upregulation. The role of SK1 is not limited to the induction of aerobic glycolysis, affecting metabolic pathways that appear to support the biosynthesis of macromolecules. These findings highlight the role of SK1 signaling axis in cancer metabolic reprogramming, pointing out innovative strategies for cancer therapies.

**Abbreviations**
Cer: ceramide
CPMG: Carr–Purcell–Meiboom–Gill
G6PDH: glucose‐6‐phosphate dehydrogenase
HBP: hexosamine biosynthesis pathway
HPLC: high‐performance liquid chromatography
HPR: N‐(4‐hydroxyphenyl) retinamide
IMP: inosine monophosphate
M‐PLS: multilevel partial least squares
MS: mass spectrometry
PCA: principal component analysis
PLS: partial least squares
PPP: pentose phosphate pathway
S1PR: sphingosine 1‐phosphate receptors
S1P: sphingosine 1‐phosphate
SK: sphingosine kinase
Sph: sphingosine
SPL: sphingosine 1‐phosphate lyase
SPP: sphingosine 1‐phosphate phosphatase
TCA: tricarboxylic acid cycle
UDP‐GlcNAc: uridine diphosphate *N*‐acetylglucosamine
WB: western blotting

## 1 Introduction

Sphingosine kinase (SK) catalyzes the ATP‐dependent phosphorylation of sphingosine (Sph) leading to the production of the bioactive sphingolipid sphingosine 1‐phosphate (S1P) (Pyne *et al*., 2016). Two different isoforms of SK do exist, SK1 and SK2 that, although both contribute to intracellular S1P formation, have been found to display opposite biological roles due to their different subcellular localization and regulation (Maceyka *et al*., 2005). Strong experimental evidence in animal and cellular models supports the involvement of SK1 in oncogenesis (Heffernan‐Stroud and Obeid, 2013): this enzyme plays important roles in most tumorigenic processes being required for the promotion of survival (Vadas *et al*., 2008), proliferation (Sukocheva *et al*., 2006), transformation (Xia *et al*., 2000) as well as neoangiogenesis, which ensures tumor growth (LaMontagne *et al*., 2006).

In many human cancers such as breast, ovary, prostate, and lung cancer, SK1 levels are upregulated (Akao *et al*., 2006; Johnson *et al*., 2005; Ruckhaberle *et al*., 2008; Sutphen *et al*., 2004), and these increases are linked to poor prognosis in patients (Kim *et al*., 2015; Ruckhaberle *et al*., 2008; Van Brocklyn *et al*., 2005). Fifteen years ago, the oncogenic potential of SK1 was proposed (Xia *et al*., 2002); however so far, no mutations on *Sphk1* gene have been identified in human tumors (Vadas *et al*., 2008). The reliance of cancer cells on SK1 signaling pathway, which becomes overexpressed without any evident mutations, is called nononcogene addition (Vadas *et al*., 2008). The exact molecular mechanisms that lead to the addition of cancer cells to SK1 signaling pathway and the precise mechanism of action of SK1 in cancer development remain to be elucidated.

Elevated levels of SK1 have also been shown to contribute to pancreatic cancer cell resistance to gemcitabine and chronic myeloid leukemia resistance to imatinib (Pyne *et al*., 2011). In this regard, we reported that in A2780 human ovarian carcinoma cells resistant to the synthetic retinoid N‐(4‐hydroxyphenyl) retinamide (HPR) SK1 activity, mRNA and protein levels were increased and treatment with an SK inhibitor resulted in increased sensitivity to HPR (Illuzzi *et al*., 2010). Moreover, the overexpression of SK1 in A2780 cells was sufficient to induce resistance to HPR, demonstrating the key role of SK1 in determining the resistance to the chemotherapeutic drug (Illuzzi *et al*., 2010).

The catabolism of the bioactive lipid S1P is under the control of different enzymes: S1P can be irreversibly degraded by S1P lyase (SPL) or reversibly dephosphorylated to Sph by two distinct specific S1P phosphatases (SPP), which can be then acylated to ceramide (Cer) in the sphingolipid salvage pathway (Maceyka *et al*., 2012). Cer and S1P are interconvertible lipids, and it has been proposed that their relative intracellular levels dictate the cell fate. Indeed, according to the so‐called sphingolipid rheostat, shifting the equilibrium toward S1P induces survival, while Cer increases apoptosis (Newton *et al*., 2015). S1P can be exported outside the cells and becomes the ligand, in an autocrine/paracrine manner, of a family of G protein‐coupled receptors named S1P receptors (S1PR), S1P_1‐5_, which mediate the majority of S1P effects (Blaho and Hla, 2014).

The metabolic characteristics of cancer cells are profoundly different from those of normal cells. Cancer cells are dependent on aerobic glycolysis, a metabolic feature known as Warburg effect, that explains how faster glycolytic breakdown is preferred to mitochondrial oxidative phosphorylation for energy production (Vander Heiden *et al*., 2009). Moreover, it is supposed that this cancer‐related metabolic adaptation is necessary to facilitate the incorporation of nutrients such as nucleotides, amino acids, and lipids into the biomass required for enhanced cancer cell proliferation (Hsu and Sabatini, 2008). This metabolic cancer reprogramming has been linked also to therapeutic resistance in cancer (Zhou *et al*., 2010). Indeed, targeting cancer cell metabolism in combination with chemotherapeutic drugs may represent a promising approach to inhibit cancer growth and overcome drug resistance. However, a better understanding of the molecular mechanisms and cellular targets that link metabolic regulation with signaling pathways involved in the modulation of survival, proliferation, and drug resistance of cancer cells is required.

Here, by employing an untargeted NMR metabolomic profiling combined with functional validations, we show for the first time that SK1 plays a crucial role in the metabolic reprogramming of A2780 human ovarian cancer cells. Indeed, stable overexpression of SK1 induced high glycolytic rate, with increased glucose uptake and augmented levels of lactate, and decreased oxidative metabolism, associated with accumulation of intermediates of the tricarboxylic acid cycle (TCA) and reduction in CO_2_ production. Furthermore, the reported data show that the role of SK1 is not restricted to the aerobic glycolysis switch, affecting metabolic pathways that may support synthesis of macromolecules.

These results point out novel molecular mechanisms for cancer development and progression and potential innovative strategies in cancer therapy.

## 2 Materials and methods

### 2.1 Cell culture and transfection

Cells were cultured in RPMI 1640 supplemented with 10% of heat‐inactivated fetal bovine serum, 2 mm glutamine, 100 units·mL^−1^ penicillin, and 100 μg·mL^−1^ streptomycin. A2780 cells were transfected by FuGENE (Roche Applied Science, Mannheim, Germany) with the pcDNA3‐hSK1^WT^FLAG (Pitson *et al*., 2000) (a gift from Stuart M. Pitson, Centre for Cancer Biology, Adelaide, Australia) or with the empty vector, following the manufacturer's protocol. A mixed population of transfected drug‐resistant cells were isolated after selection with 500 μg·mL^−1^ geneticin (Sigma‐Aldrich, St. Louis, MO, USA) for three weeks. Under these selective conditions, a polyclonal population of stably expressing cells was obtained. The selected stable transfectants were then cultured in the presence of 250 μg·mL^−1^ geneticin.

### 2.2 NMR sample preparation

Four independent experiments were performed. In each experiment, mock‐ and SK1‐expressing cells were plated and grown until subconfluence. The media of two subconfluent p100 were replenished with fresh media (FBS: growing condition; without FBS: serum starvation condition) for 24 h prior to cell harvesting. This allowed for standardization of the time between equilibration of extra‐ and intracellular metabolites and reduced the effects of substrate limitation on the intracellular metabolism. Metabolomic studies were performed in growing cells and in serum‐starved cells (RPMI 1640 supplemented with 1 mg·mL^−1^ BSA, 2 mm glutamine, 100 units·mL^−1^ penicillin, and 100 μg·mL^−1^ streptomycin). After 24 h, the media were removed and the cells washed twice with ice‐cold PBS, dispersed in a buffer solution containing 10 mm Tris pH 7.4, 5 mm EDTA, 120 mm NaCl, protease inhibitor [1 mm 4‐(2‐aminoethyl)benzenesulfonyl fluoride, 0.3 μm aprotinin, 10 μg·mL^−1^ leupeptin, and 10 μg·mL^−1^ pepstatin], and phosphatase inhibitors (Phosphatase Inhibitor Cocktail 3; Sigma‐Aldrich). Cell lysis was performed by sonication and the cytosolic fraction containing the metabolites was obtained as previously described (Bernacchioni *et al*., 2012; Cencetti *et al*., 2010, 2013).

From each of the four independent experiments, we obtained four samples: mock growing; SK1 growing; mock serum‐starved; and SK1 serum‐starved. NMR samples were prepared in 5.00 mm NMR tubes (Bruker BioSpin srl, Rheinstetten, Germany) after the addition of 50 μL of ^2^H_2_O containing 10 mm sodium trimethylsilyl [2,2,3,3‐^2^H_4_]propionate to 450 μL of cell lysate.

### 2.3 Cell treatment

Three to five independent experiments were performed for each treatment reported below.

In order to investigate the effect of exogenously added S1P on A2780 cell metabolism, subconfluent mock cells were treated with 1 μm S1P for 24 h under serum starvation.

To evaluate the involvement of S1P_3_ in the metabolic shift induced by SK1 or the effect of SK1 inhibition, subconfluent SK1‐expressing cells were treated under serum starvation with the S1P_1/3_ antagonist VPC23019 (1 μm) for 24 h or the SK1‐specific inhibitor VPC96091 (5 μm) for 24 h, respectively.

### 2.4 NMR experiments

^1^H NMR spectra were acquired on cell lysates verifying their feasibility and reproducibility (Fig. S1). NMR spectra were recorded with a Bruker 900‐MHz spectrometer equipped with CP TCI ^1^H/^13^C/^15^N probe. ^1^H NMR spectra were acquired with the Carr–Purcell–Meiboom–Gill (CPMG) sequence using a monodimensional spin–echo sequence with water presaturation (cpmgpr; Bruker BioSpin srl); 128 scans over a spectral region of 18 kHz were collected into 110 K points, giving an acquisition time of 3.07 s. The CPMG pulse sequence (Carr and Purcell, 1954) was used to impose a T_2_ filter that allows selective observation of small molecular weight components. The total T_2_ delay was set to 290 ms. The T_2_ filtering in the CPMG pulse sequence, which contains trains of ‐(τ‐180°‐τ)‐ blocks repeated *n *=* *128 times, was achieved with a total spin echo delay (2nτ) of 80 ms. The acquisition of each spectrum required about 16 min. The raw data were multiplied by a 1‐Hz exponential line broadening before Fourier transformation into 131 K points. Transformed spectra were automatically corrected for phase and baseline distortions and calibrated (chemical shift was referenced to the proton of alanine at δ 1.48 p.p.m.) using TopSpin 3.5 (Bruker BioSpin srl). We successfully performed this type of experiments on samples obtained from independent experiments identifying the metabolomic fingerprint of A2780 parental cells and of their derivative A2780 cells overexpressing SK1, as well as of cells treated with S1P, VPC96091, and VPC23019.

### 2.5 Spectral analysis

Each spectrum in the region 10.00–0.2 p.p.m. was segmented into 0.02 p.p.m. chemical shift bins, and the corresponding spectral areas were integrated using the amix software (Bruker BioSpin srl). The area of each bin was normalized to the total spectral area (Craig *et al*., 2006; Smolinska *et al*., 2012), calculated with exclusion of the regions 2.60–2.83, 3.23–3.28, 3.35–3.39, 3.63–3.75, 6.45–6.49, 7.30–7.36, 8.44–8.48 (which correspond to EDTA peaks and the most intense peaks of the protease and phosphatase inhibitors used for sample preparation) and 4.2–5.9 p.p.m. (water region).

### 2.6 Metabolite statistical analysis

Various kinds of multivariate and univariate statistical techniques were applied on the obtained buckets using R 3.0.2 in house scripts.

Unsupervised principal component analysis (PCA) was used to obtain a preliminary outlook of the data (visualization in a reduced space, clusters detection, screening for outliers). From the PCA score plot (PC1, PC2, PC3), Fig. S2, a perfect discrimination between the two different conditions tested (growing and serum starvation) was clearly visible, indicating a strong influence on the cell metabolic profiles; nevertheless, a great discrimination between mock cells and SK1‐expressing cells was clearly visible in both conditions tested. Partial least squares (PLS) was employed to perform supervised data reduction and classification between growing and the serum starvation condition. In order to analyze the difference in the metabolic fingerprint induced by SK1 expression, multilevel partial least squares (M‐PLS) was employed to perform supervised data reduction and classification. With this approach, within each independent experiment, for a specific experimental condition, mock‐ and SK1‐expressing cells can be considered as paired control and treated samples, thus allowing the reduction in the experimental variability between independent cell culture experiments and the metabolic variability introduced by the media composition (van Velzen *et al*., 2008; Westerhuis *et al*., 2010). The global accuracy for classification was assessed by means of a leave‐one‐out cross‐validation scheme.

Thirty‐two metabolites, whose peaks in the spectra were well defined and resolved, were assigned and their levels analyzed (Table S1), after total area normalization calculated as described in Section 2.4. The assignment procedure was performed using a NMR spectra library of pure organic compounds, public databases (e.g., Human Metabolome Database), storing reference NMR spectra of metabolites, spiking NMR experiments, and using literature data. The relative concentrations of the various metabolites were calculated by integrating the corresponding signals in the spectra (Wishart, 2008). The paired Wilcoxon test was used for the determination of the meaningful metabolites: a *P*‐value of 0.05 was considered statistically significant. The concentration changes in the metabolites are also expressed as the log_2_(FC) (fold change; Fig. S3).

### 2.7 Sphingolipid mass spectrometry (MS)

Sph and S1P were extracted as recently described (Japtok *et al*., 2015; Pewzner‐Jung *et al*., 2014) using D7‐S1P and D7‐Sph as internal standards. Sphingolipids were separated by reverse‐phase high‐performance liquid chromatography (HPLC) (Agilent 1290 series; Agilent Technologies, Waldbronn, Germany) using a ZORBAX Eclipse Plus C8 column (2.1 × 150 mm, ID: 3.5 μm) (Agilent Technologies). Solvent A was 50 : 50 methanol/acetonitrile with 0.1% formic acid and solvent B was water with 0.1% formic acid. The gradient was held at 60% A between 0 and 3 min, increased linearly from 60% A to 84% A between 3 min and 11 min, returned linearly to 60% A and held for 3 min to allow column re‐equilibration. The flow rate was 0.5 mL·min^−1^. The injection volume was 10 μL. The HPLC column effluent was introduced onto a QQQ 6490 mass spectrometer (Agilent Technologies) operating in the positive ESI mode. Capillary and V charging were 4.5 and 2 kV, respectively. Drying gas temperature was 290 °C, drying gas flow rate was 11 L·min^−1^, sheath gas temperature was 380 °C, sheath gas flow rate was 12 L·min^−1^, and nebulizer pressure was 35 psi. The precursor ion of Sph *m*/*z* 300.29 was cleaved into the fragment ion of *m*/*z* 282.3 at 8 eV; the precursor ion of D7‐Sph *m*/*z* 307.3 was cleaved into the fragment ion of *m*/*z* 289.3 at 8 eV. The precursor of S1P *m*/*z* 380.26 was cleaved into the fragment ion of *m*/*z* 264.3 at 16 eV. The precursor of D7‐S1P *m*/*z* 387.3 was cleaved into the fragment ion of *m*/*z* 271.3 at 16 eV. Ceramides were extracted and quantified as recently described *(*Huston *et al*., 2016 *)*. Briefly, lipid extraction was performed using C17‐Cer as internal standard. Sample analysis was carried out by HPLC‐MS/MS using a Q‐TOF 6530 mass spectrometer (Agilent Technologies) operating in the positive ESI mode. The precursor ions of ceramides (C16‐Cer (*m*/*z* 520.508), C17‐Cer (*m*/*z* 534.524), C18‐Cer (*m*/*z* 548.540), C20‐Cer (*m*/*z* 576.571), C22‐Cer (*m*/*z* 604.602), C24‐Cer (*m*/*z* 632.634), C24:1‐Cer (*m*/*z* 630.618)) were cleaved into the fragment ion of *m*/*z* 264.270. Quantification was performed with mass hunter Software (Agilent Technologies).

### 2.8 Glucose uptake

Glucose uptake was evaluated in a buffered solution (140 mm NaCl, 20 mm Hepes/Na, 2.5 mm MgSO_4_, 1 mm CaCl_2_, and 5 mm KCl, pH 7.4) containing 0.5 μCi·mL^−1^ [U‐^14^C] glucose (Perkin Elmer, Waltham, MA, USA) for 15 min at 37 °C. Cells were subsequently washed with cold PBS and lysed with 0.1 m NaOH. Incorporated radioactivity was assayed by liquid scintillation counting and normalized on protein content as previously described (Rapizzi *et al*., 2009).

### 2.9 Detection of released CO_2_ by radioactive glucose

A2780 cells were added with 0.5 μCi·mL^−1^ [U‐^14^C] glucose (Perkin Elmer) for 15 min. Each dish had a taped piece of Whatman paper facing the inside of the dish wetted with 100 μL of phenyl‐ethylamine/methanol (1 : 1) to trap CO_2_. Then, 200 μL of 4 m H_2_SO_4_ was added to cells. Whatman paper was removed and transferred to scintillation vials for counting. Radioactive signal was measured by liquid scintillation counting and normalized for protein content.

### 2.10 Quantitative real‐time reverse transcription PCR

Total RNA (2 μg), extracted with TRI reagent from A2780 cells, was reverse‐transcribed using the high‐capacity cDNA reverse transcription kit (Applied Biosystems, Foster City, CA, USA). The quantification of S1P metabolism enzymes (SK1, SK2, SPL, SPP1, and SPP2) and S1PR mRNA was performed by real‐time PCR employing TaqMan gene expression assays. Each measurement was taken in triplicate, using the automated ABI Prism 7500 Sequence Detector System (Applied Biosystems) as described previously (Cencetti *et al*., 2013; Donati *et al*., 2011), by simultaneous amplification of the target sequence together with the housekeeping gene β‐actin. Results were analyzed by ABI Prism sequence detection system software, version 1.7 (Applied Biosystems). The 2^−ΔΔCT^ method was applied as a comparative method of quantification (Livak and Schmittgen, 2001), and data were normalized to β‐actin RNA expression.

### 2.11 Western blot analysis

A2780 cells lysates were obtained as described in the NMR sample preparation section. Proteins from cell lysates were resuspended in Laemmli's SDS sample buffer. Samples were subjected to SDS/PAGE and western blot analysis. Bound antibodies were detected using ECL reagents (Bernacchioni *et al*., 2011; Gangoiti *et al*., 2012). The intensities of the detected specific bands were normalized on the band intensities of the loading controls (β‐actin or total ERK or total Akt), and the resulted data were reported as mean ± SEM, ‐fold change over control set as 1.

## 3 Results and Discussion

### 3.1 Study design

Our previous results demonstrated that SK1 expression in A2780 human ovarian cancer cells is sufficient to induce resistance to the chemotherapeutic agent HPR (Illuzzi *et al*., 2010). To understand the cellular processes induced by SK1 expression, here we focused on SK1 regulation of cancer cell metabolism. The experimental plan involved two different conditions: (i) the growing condition, in order to characterize the differences induced by SK1 expression in ovarian cancer cells, and (ii) the serum starvation condition, to mark out the effect of SK1 expression in the absence of mitogens. In order to study the metabolic phenotype, we propose a multidisciplinary approach based on matching molecular/cellular biology methods with NMR‐based metabolomics, an untargeted method to determine the overall intracellular metabolite composition pool, readout of any metabolic activity, called metabolome.

### 3.2 SK1 expression in ovarian cancer cells affects the metabolomic profiles

**Figure 1:**
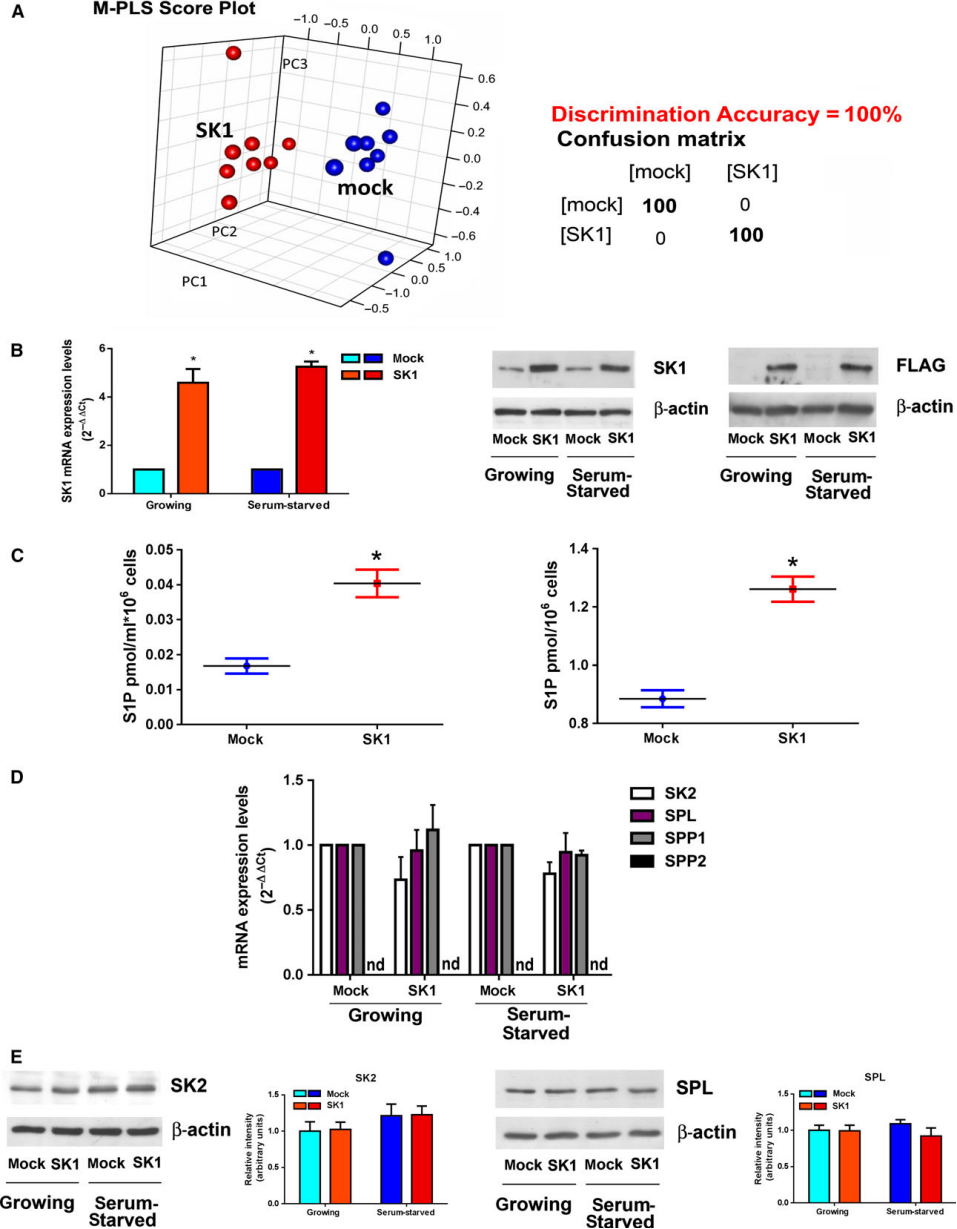
SK1 overexpression in A2780 ovarian cancer cells. (A) Score plot of multilevel PLS discrimination of metabolomics profiles of mock‐ (blue dots) and SK1‐ (red dots) cell lysates. The plot was constructed using ^1^H‐NMR CPMG spectra. Right panel: cross‐validation paired test and confusion matrix for mock and SK1 samples; sensitivity of the test = 100%; specificity of the test = 100%. (B) Overexpression of SK1 tagged with FLAG M2 epitope. Left panel: quantitative analysis of SK1 mRNA by real‐time RT‐PCR as fold changes according to the 2^−∆∆CT^ method. Data are reported as mean ± SEM of three independent experiments. The overexpression of SK1 in pcDNA3‐hSK1^WT^‐transfected compared to empty vector‐transfected cells was statistically significant by Student's *t*‐test, *P < 0.05. Central and right panels: SK1‐FLAG expression levels in stably transfected A2780 cells by WB analysis using specific monoclonal anti‐SK1 (central panel) and anti‐FLAG (right panel) antibodies. Equally loaded protein was checked by expression of β‐isoform of actin. (C) Mock‐ and SK1‐expressing cells were serum‐starved for 24 h before the media were collected (left panel) and cells harvested (right panel) and then subjected to S1P analysis. The effect of SK1 expression on S1P levels in the media and inside the cells was statistically significant by Student's *t*‐test *P < 0.05. (D) Quantitative mRNA analysis by real‐time PCR in total extracted RNA. SK2, SPL, SPP1, and SPP2 mRNA quantitation was based on the 2^−∆∆CT^ method, using SK2, SPL, or SPP1 of the mock‐transfected specimen as calibrator. Data are the mean ± SEM of three experiments performed in triplicate. nd, not detected. (E) SK2 and SPL expression by WB analysis. Equally loaded protein was checked by expression of β‐isoform of actin. A blot representative of three independent experiments with analogous results is shown. Bar plots represent the densitometric analysis of at least three independent experiments. Data are the mean ± SEM and are reported as protein expression normalized to β‐actin, ‐fold change over control set as 1.

In order to study the possible metabolomic changes induced by SK1 expression, ^1^H‐NMR spectra were acquired (Fig. S1) using a high‐field 900‐MHz spectrometer equipped with cryoprobe. From the acquired NMR profiles of growing and serum‐starved A2780 cells, it emerges that there are obvious differences (attributable to metabolic depression under starvation), which can be discriminated at the level of unsupervised PCA (Fig. S2), and have a discrimination accuracy of 100% using a PLS supervised approach. Nevertheless, we could successfully identify the metabolomic fingerprint of A2780 ovarian cancer parental cells as well as their derivative overexpressing SK1, independently on the growing conditions. Using an NMR‐untargeted approach, we demonstrated that SK1 expression is sufficient to alter radically the metabolomic profile of ovarian cancer cells (Fig. 1A), with a discrimination accuracy of 100% between SK1‐expressing A2780 and control mock cells when a M‐PLS statistical analysis was applied.

Stable overexpression of SK1 in A2780 human ovarian cells was verified through mRNA relative quantification by real‐time RT‐PCR analysis and by measuring protein expression levels through western blotting (WB) (Fig. 1B). Consistently, S1P levels, as measured by MS, increased significantly in both lysates and media of SK1‐expressing cells with respect to mock control cells (Fig. 1C). In accordance, we observed a significant decrease in Sph levels, while no significant changes were observed in terms of Cer levels (Fig. S4). We then determined whether other S1P‐metabolizing enzymes were altered because of SK1 expression. No compensatory alterations on the expression levels of SK2 occurred, as shown by real‐time RT‐PCR analysis (Fig. 1D) and WB (Fig. 1E). Accordingly, the involvement of SK2 isoform in the metabolic shift toward aerobic glycolysis was also excluded in a previous report: by employing SK inhibitors and a LC/MS approach, Watson and collaborators reported that while SK1 induces the Warburg effect on androgen‐sensitive LNCaP prostate cancer cells, SK2 exhibits a nonoverlapping function (Watson *et al*., 2013).

It has been proposed that loss of the expression of SPL, the enzyme that irreversibly degrades S1P, might contribute to tumorigenesis (Bandhuvula and Saba, 2007). Here, we analyze the effect of SK1 on the expression of SPL and S1P phosphatases in A2780 ovarian cancer cells. SPL mRNA and protein levels were not altered by SK1 expression as demonstrated by real‐time RT‐PCR analysis (Fig. 1D) and WB (Fig. 1E). Moreover, real‐time RT‐PCR analysis showed that the expression of SPP1 and SPP2, the specific S1P phosphatases, was not altered (Fig. 1D): SPP1 was not affected consequently to SK1 expression, whereas the expression of SPP2 was not detectable either in control or in SK1‐overexpressing A2780 ovarian cancer cells. Thus, it appears that enhanced SK1 expression did not overtly affect the other S1P‐metabolizing enzymes.

### 3.3 SK1 induces the Warburg effect in ovarian cancer cells

**Figure 2:**
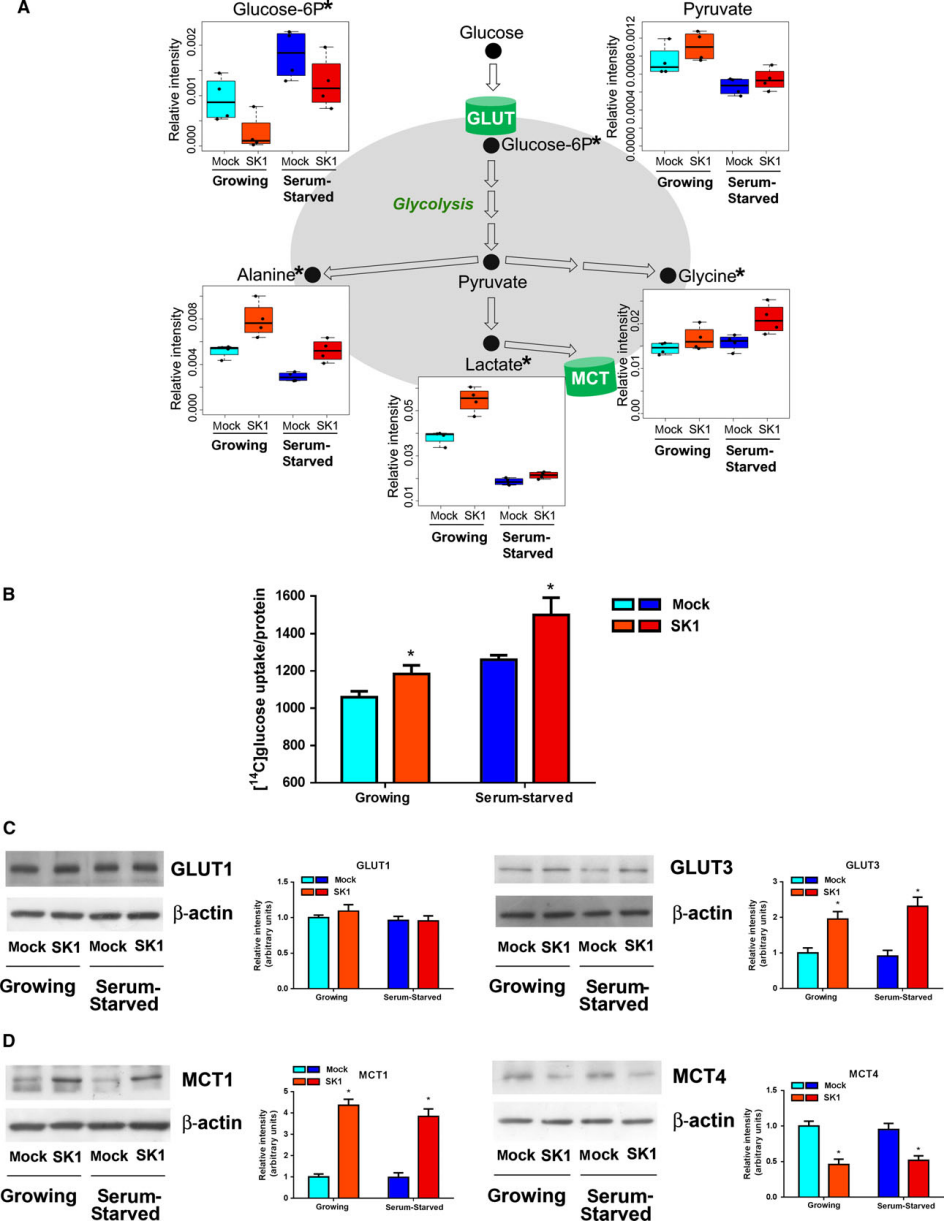
SK1 expression induces a glycolytic switch in A2780 ovarian cancer cells. A2780 mock and SK1 cells were cultured in growing medium or serum‐starved for 24 h. (A) Box plots: the relative concentration levels of the indicated metabolites in each group were calculated by integrating the signal area in the respective ^1^H‐NMR spectra. Changes in metabolite levels caused by SK1 overexpression were statistically significant by paired Wilcoxon test, *P < 0.05. (B) [^14^C]‐glucose uptake normalized on protein content. The increase in glucose uptake induced by SK1 expression was statistically significant by Student's *t*‐test, *P < 0.05. (C) Expression levels of GLUT1 and GLUT3 transporters by WB. Equally loaded protein was checked by expression of β‐isoform of actin. A blot representative of three independent experiments with analogous results is shown. Bar plots represent band intensity of GLUT1 and GLUT3 normalized to β‐actin and reported as mean ± SEM of three independent experiments, ‐fold change over control. The effect of SK1 expression was statistically significant by Student's *t*‐test, *P < 0.05. (D) MCT1 and MCT4 expression by WB. Equally loaded protein was checked by expression of β‐isoform of actin. A blot representative of three independent experiments with analogous results is shown. Bar plots represent the densitometric analysis of at least three independent experiments. Data are the mean ± SEM and are reported as protein expression normalized to β‐actin, ‐fold change over control (set as 1). The effect of SK1 expression was statistically significant by Student's *t*‐test, *P < 0.05.

Metabolomic profiles here analyzed provided steady‐state levels of metabolites, resulting from the balance between production and consumption of each molecule. The comparison of the metabolic profiles characteristic of SK1‐expressing ovarian cancer cells with those of parental cells led to the identification of SK1‐dependent significant changes in the cell metabolome. The main metabolites responsible for the discrimination are related to the glycolytic pathway. In SK1‐expressing cells, a significant decrease in glucose 6‐phosphate levels was accompanied by a marked increase in lactate levels (Fig. 2A), suggesting the occurrence of SK1‐induced Warburg effect. Consistently, the SK1‐expressing cells showed a 20% increase in the uptake of radioactive glucose (Fig. 2B). Therefore, the higher amount of uptaken glucose (Fig. 2B) is metabolized faster in SK1‐expressing ovarian cancer cells leading to lower intracellular glucose 6‐phosphate levels (Fig. 2A). Hence, SK1‐expressing ovarian cancer cells show accelerated glycolytic metabolism and high glucose requirements. The upregulation of specific glucose transporters, especially the GLUT1 and GLUT3 proteins, is reported as a key mechanism by which malignant cells achieve increased glucose uptake to support their high rate of glycolysis (Szablewski, 2013). Thus, we analyzed the expression of the glucose transporters GLUT1 and GLUT3 by WB, demonstrating that in SK1‐expressing cells GLUT3 is upregulated, while GLUT1 expression is unaffected (Fig. 2C). These results demonstrated that the increased glucose uptake positively correlates with an enhanced expression of GLUT3, suggesting the involvement of this low K_M_ transporter isoform in glucose internalization. Accordingly, GLUT3 has been found overexpressed in different types of cancer (Szablewski, 2013), including ovarian cancer (Younes *et al*., 1997).

The analysis of other metabolites directly or indirectly produced by the glycolytic pathway provided some hints on the overall process. The pyruvate levels were not significantly affected by SK1 expression. Although efficient glycolysis obviously produced high amount of pyruvate, this molecule is also the substrate of a number of biochemical processes that maintains its steady‐state levels unaffected. Indeed, besides being reduced to lactate, pyruvate can be transaminated to produce alanine, whose levels were also increased in SK1‐expressing metabolomic NMR profiles (Fig. 2A). A significant portion of glycolytic carbon can be diverted toward serine and glycine biosynthesis by phosphoglycerate dehydrogenase. Consistently, glycine levels were also increased (Fig. 2A). Altogether, these results demonstrate that SK1 expression induces a significant increase in glycolysis under aerobic conditions.

The glycolytic phenotype results in increased lactate production. Consequently, to prevent cellular acidosis, tumor cells display an increased expression of the proton/monocarboxylate symporters MCT. Because of the relevance of MCT1 and/or MCT4 as hallmarks of several human malignancies (Halestrap and Meredith, 2004; Halestrap and Wilson, 2012), here we evaluated their expression levels by WB. As shown in Fig. 2D, SK1‐expressing ovarian cancer cells displayed a significant increase in MCT1 while MCT4 was downregulated, suggesting a major role for MCT1 in driving the efflux of proton/lactate in these cells. Accordingly, MCT1 recently emerged as a key promoter of drug resistance in ovarian cancer by antagonizing Fas (Yan *et al*., 2015). Moreover, MCT1 strongly increased in both cisplatin‐resistant ovarian cancer tissue and cell lines compared with sensitive ovarian cancer tissue and cells, respectively. Furthermore, in gastric cancer, a significant downregulation of MCT4 has been correlated with the malignant progression (Pinheiro *et al*., 2009).

### 3.4 SK1 affects the tricarboxylic acid cycle (TCA)

**Figure 3:**
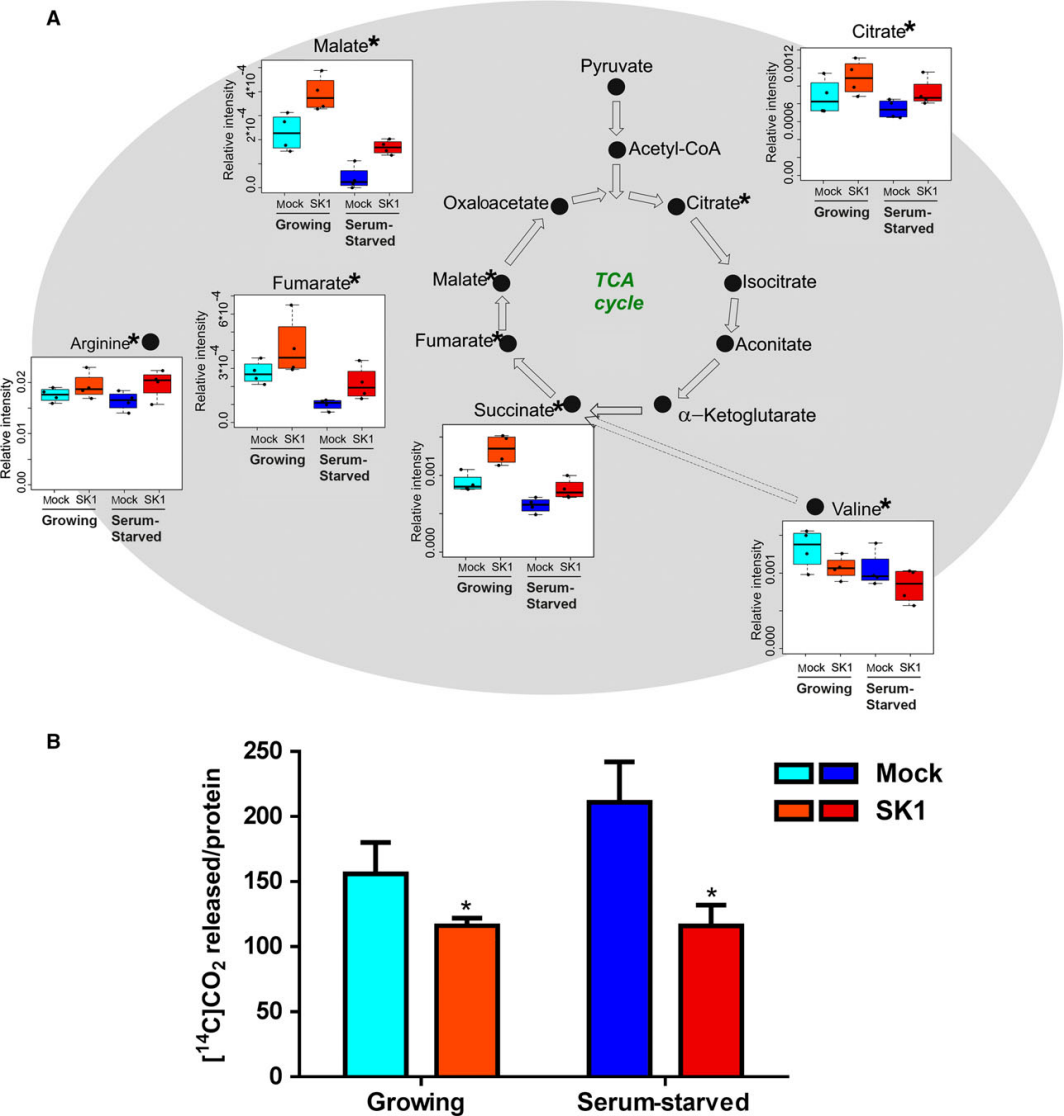
SK1 expression reduces oxidative metabolism in A2780 ovarian cancer cells. A2780 mock and SK1 cells were cultured in growing medium or serum‐starved for 24 h. (A) Box plots: the relative concentration levels of the indicated metabolites in each group were calculated by integrating the signal area in the respective ^1^H‐NMR spectra. Changes in metabolite levels caused by SK1 expression were statistically significant by paired Wilcoxon test, *P < 0.05. (B) Respiration of [^14^C]‐glucose was evaluated as [^14^C]‐CO_2_ release. The reduction in [^14^C]‐CO_2_ release caused by SK1 expression was statistically significant by Student's *t*‐test, *P < 0.05.

Untargeted metabolomic profiles also highlighted a significant increase in the intracellular levels of citrate, succinate, fumarate, and malate in SK1‐expressing cells under aerobic condition (Fig. 3A). Consistently, SK1‐expressing cells displayed a significant reduction in CO_2_ production (Fig. 3B).

These results suggest that SK1 expression altered the TCA cycle with consequent accumulation of reaction intermediates. The strong increase in these intermediate levels could be, at least partially, ascribed to feeding pathways. Accordingly, the here observed SK1‐induced significant reduction in the essential amino acid valine (Fig. 3A) can be tentatively ascribed to an enhanced valine catabolism brought about to support the TCA cycle at the level of succinate via propionyl‐CoA and succinyl‐CoA formation; nevertheless, the quantification of more intermediates would be necessary to confirm the involvement of this pathway. Notably, it has been recently proposed that fumarate and succinate behave as oncometabolites that, upon accumulation, become responsible for different malignant‐associated states (Yang *et al*., 2013).

From the NMR profiles, we measured an SK1‐induced increase in the levels of arginine (Fig. 3A), a semi‐essential amino acid (Appleton, 2002) critical for the growth of human cancers. The *de novo* synthesis of arginine relies on the enzyme argininosuccinate synthase, expressed in ovarian cancer (Nicholson *et al*., 2009), that leads to the formation of arginine and fumarate, one of the TCA intermediates found augmented consequently to SK1 expression (Fig. 3A). It is interesting to note that the increase in arginine levels is relevant *per se* because, besides being used for protein synthesis, this amino acid is involved in multiple aspects of tumor metabolism, including the synthesis of nitric oxide, polyamines, nucleotides, proline, and glutamate (Delage *et al*., 2010).

In order to further underline the specificity of the metabolic changes caused by SK1 activity, SK1‐expressing A2780 cells were treated with a specific SK1 inhibitor before being processed for the NMR‐based metabolomic analysis. The metabolic profiles obtained from SK1‐expressing A2780 cells incubated with the SK1‐specific inhibitor VPC96091 (5 μm for 24 h) showed that the changes in glycolytic and TCA metabolite levels induced by SK1 expression were strongly reduced by SK1 inhibition (Fig. S5). In summary, these data definitely demonstrate that increased SK1 protein level and activity in ovarian cancer cells is causative of high glycolytic rates and decreased oxidative metabolism under aerobic conditions. Therefore, SK1 positively regulates glycolysis for bioenergetic demand.

### 3.5 Dysregulation of other metabolic pathways in SK1‐expressing ovarian cancer cells

**Figure 4:**
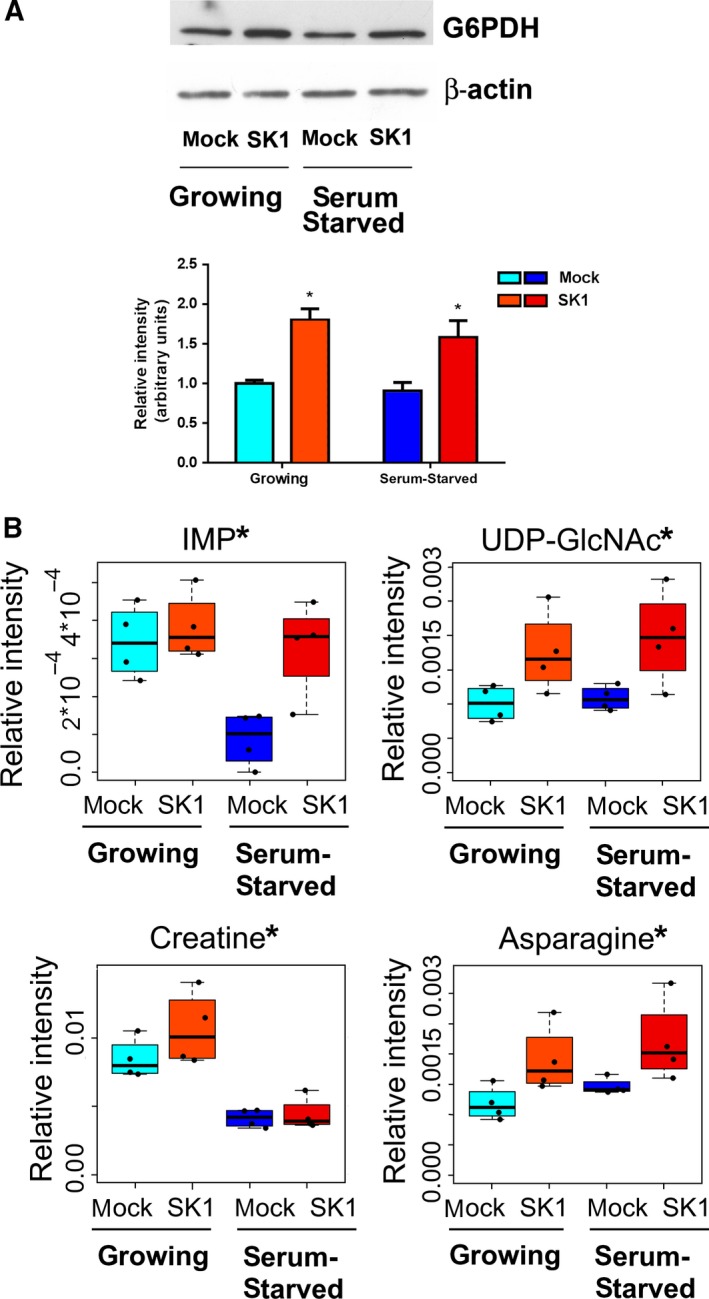
SK1‐induced metabolic changes in A2780 ovarian cancer cells. A2780 mock and SK1 cells were cultured in growing medium or serum‐starved for 24 h. (A) G6PDH expression by WB. Equally loaded protein was checked by expression of β‐isoform of actin. A blot representative of three independent experiments with analogous results is shown. The bar plot represents band intensity of G6PDH normalized to β‐actin and reported as mean ± SEM of three independent experiments, ‐fold change over control. The effect of SK1 expression was statistically significant by Student's *t*‐test, *P < 0.05. (B) Box plots: the relative concentration levels of the indicated metabolites in each group were calculated by integrating the signal area in the respective ^1^H‐NMR spectra. Changes in metabolite levels caused by SK1 expression were statistically significant by paired Wilcoxon test, *P < 0.05.

High‐proliferating cancer cells must not only generate enough energy to support cell replication, but also satisfy the anabolic demands of macromolecular biosynthesis and maintain cellular redox homeostasis. The variation observed in some metabolites or enzymes whose levels changed significantly in SK1‐expressing cells is discussed below in the context of the implicated pathways. Nevertheless, the interpretation of their role remains at the level of hypothesis due to the lack of information on the concentration of more intermediates of the identified pathways. The pentose phosphate pathway (PPP) is a major glucose catabolic pathway that supplies anabolism‐linking glucose to the biosynthesis of the nucleotide precursor ribose and to NADPH production. This latter process is essential for both antioxidant defense and reductive biosynthesis, such as fatty acid synthesis. PPP is reported to be augmented by oncoproteins (Jiang *et al*., 2014). The first enzyme in the PPP pathway is glucose 6‐phosphate dehydrogenase (G6PDH), which catalyzes the dehydrogenation of glucose 6‐phosphate through an irreversible, rate‐limiting reaction. The results of WB analysis, shown in Fig. 4A, demonstrate that SK1 expression determined an increase in G6PDH expression levels in ovarian cancer.

Glycolysis and PPP are two metabolic pathways that are tightly connected and cooperatively regulate glucose uptake and metabolism. SK1 expression was sufficient to modulate both pathways in ovarian cancer cells, providing further experimental support to the significant reduction in intracellular glucose 6‐phosphate levels (Fig. 2A).

An increased SK1‐induced biosynthesis of nucleotides is consistent with the increased levels of inosine monophosphate (IMP), as shown in Fig. 4B. IMP represents the final product of purine biosynthesis: both adenine and guanine derive from IMP.

It is known that a small fraction of glucose (≤ 5%) is used in the hexosamine biosynthesis pathway (Vander Heiden *et al*., 2009). This metabolic route generates, as ultimate product, the nucleotide sugar uridine diphosphate *N*‐acetylglucosamine (UDP‐GlcNAc). Several key metabolites, including glucose 6‐phosphate from glycolysis and uridine from nucleotide biosynthesis, are required for UDP‐GlcNAc formation. Interestingly, greater increases in UDP‐GlcNAc levels correlate with higher grades of tumor development (Chaiyawat *et al*., 2014). In this regard, our metabolomic analysis showed a clear rise in UDP‐GlcNAc levels in SK1‐expressing cells (Fig. 4B).

Higher levels of creatine were found in SK1‐expressing cells (Fig. 4B). Creatine and phosphocreatine provide an intracellular, high‐energy phosphate buffering system, essential to maintain ATP levels in tissues with high‐energy demands. Although creatine synthesis from arginine and glycine primarily takes place in the kidney and liver, the expression of the enzymes involved in its synthesis has been reported for different types of cancer (Bera *et al*., 2008). Therefore, the high creatine levels observed in SK1‐expressing ovarian cancer cells could depend on its increased biosynthesis. In accordance with this hypothesis, glycine and arginine levels are also significantly augmented in consequence of SK1 expression (Figs 2A and 4B).

Moreover, asparagine, well‐known important regulator of cancer cell amino acid homeostasis, anabolic metabolism, and proliferation (Krall *et al*., 2016), is found augmented in consequence of SK1 expression in ovarian cancer cells (Fig. 4B).

### 3.6 Activated signaling pathways in SK1‐expressing ovarian cancer cells

**Figure 5:**
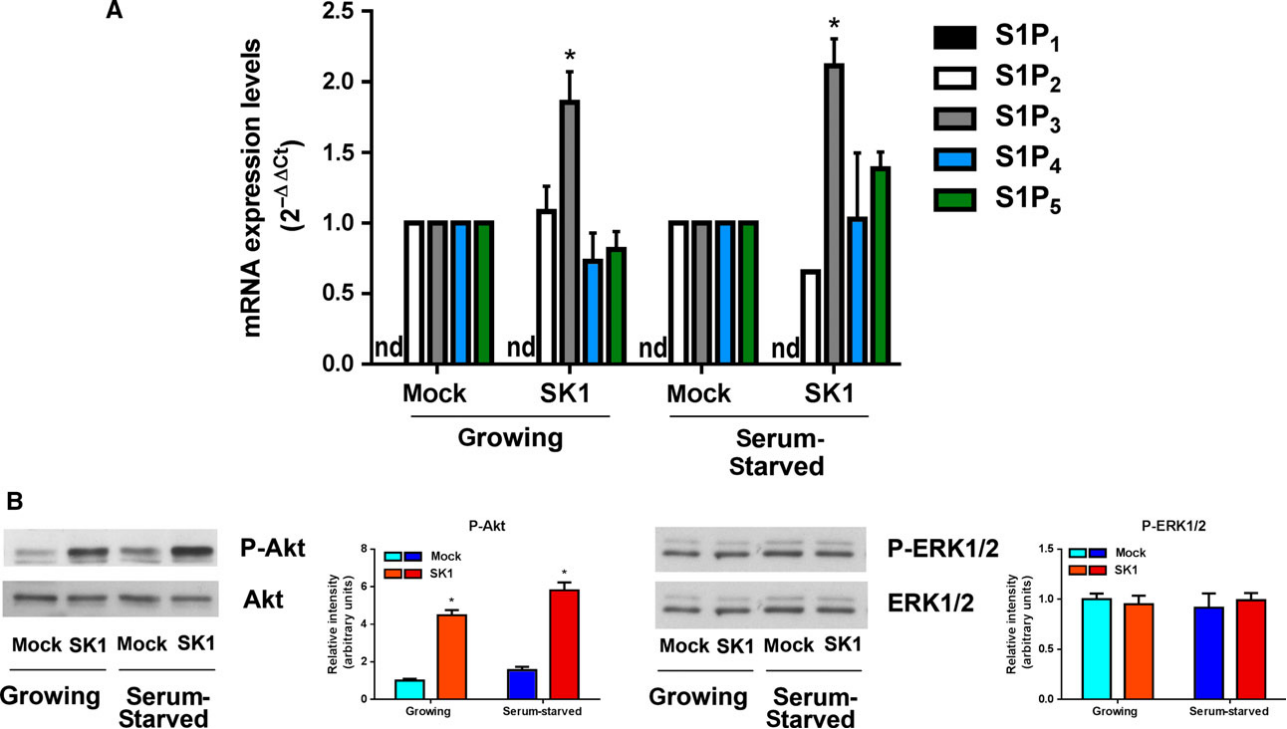
SK1‐induced pathways in A2780 ovarian cancer cells. Ovarian mock and SK1 A2780 cells were cultured in growing medium or serum‐starved for 24 h. (A) Quantitative mRNA analysis by real‐time PCR in total extracted RNA. S1PR mRNA quantitation was based on the 2^−∆∆CT^ method, using S1P_2_, S1P_3_, S1P_4_, or S1P_5_ of the mock‐transfected specimen as calibrator. nd, not detected. The increase in S1P_3_ expression in SK1‐expressing cells was statistically significant by Student's *t*‐test, *P < 0.05. (B) Cell lysates were analyzed by WB using specific anti‐phospho‐Akt, anti‐pan Akt, anti‐phospho‐ERK1/2, and anti‐pan ERK1/2 antibodies. Blots representative of at least three independent experiments are shown. Bar plots represent densitometric quantification of phosphorylated proteins normalized to their total content and reported as mean ± SEM of three independent experiments, ‐fold change over control set as 1. The effect of SK1 expression was statistically significant by Student's *t*‐test, *P < 0.05.

S1P is widely reported to act through an inside‐out signaling mechanism that occurs via export of intracellularly generated S1P and ligation to S1PR. We determined the expression levels of S1PR by mRNA relative quantification through real‐time RT‐PCR analysis. Both control and SK1‐expressing A2780 cells express four out of five S1PR, S1P_2_, S1P_3_, S1P_4_, and S1P_5_, while S1P_1_ expression was not detectable in any experimental condition (Fig. 5A). Independently from the culture conditions, the expression of SK1 caused a strong induction of S1P_3_ expression, the other receptor isoforms being unaltered, thus suggesting the existence of an amplification loop between SK1 and S1P_3_, already observed in TGF‐β‐treated myoblasts (Cencetti *et al*., 2010) and in breast cancer cells (Long *et al*., 2010). To test this hypothesis, we treated SK1‐expressing cells with VPC23019 (1 μm for 24 h), a specific antagonist for S1P_1_ and S1P_3_. In this cellular context, VPC23019 should antagonize only S1P_3_, with S1P_1_ not being expressed. Under these experimental conditions, none of the metabolite level changes measured by NMR resulted meaningful (data not shown), suggesting that this receptor subtype does not play a role in the metabolic switch induced by SK1. Nevertheless, there was a clear contribution from exogenously added S1P on A2780 cell metabolism. Indeed, S1P administration (1 μm for 24 h) to mock control A2780 cells induced a significant increase in lactate levels and a decrease in glucose 6‐phosphate levels, while concentration changes in TCA intermediates were not significant (Fig. S6). It is tempting to speculate that acute S1P administration might not be sufficient to sustain a metabolic switch, being not able to mimic continuous S1P production following SK1 expression. Moreover, the topology of endogenously produced S1P could be differently regulated with respect to exogenously added S1P (Nincheri *et al*., 2010). However, additional future studies are required to deeply characterize the role of S1PR involvement in the SK1‐induced metabolic shift.

Among the signaling pathways correlated with S1PR activation, we analyzed the activation state of two key signal transduction pathways, Akt and ERK1/2. The WB shown in Fig. 5B indicates that a sustained activation of Akt signaling is present in SK1‐expressing cells independently from the presence of mitogens in the culture medium, while ERK1/2 phosphorylation is unaffected.

Several signaling cascades are deregulated in cancer cells and appear to control altered metabolism, apoptosis, and other phenotypic features of cancer cells (Giancotti, 2014). In this context, Akt has been reported to play a key role in the metabolic conversion of cancer cells toward aerobic glycolysis in different ways. Indeed, Akt signaling has been shown to be implicated in the upregulation of glucose transporters as well as multiple glycolytic enzymes (Robey and Hay, 2009), thus increasing glucose import and consumption by cancer cells. The activation of Akt is often detected following membrane receptor(s) engagement and is implicated in different S1PR downstream signaling (Blaho and Hla, 2014), suggesting that the here observed increased phosphorylation of Akt may rely on the engagement of S1PR through SK1‐derived S1P, according to the well‐established inside‐out mechanism of action of the bioactive sphingolipid (Takabe *et al*., 2008).

## 4 Conclusions

**Figure 6:**
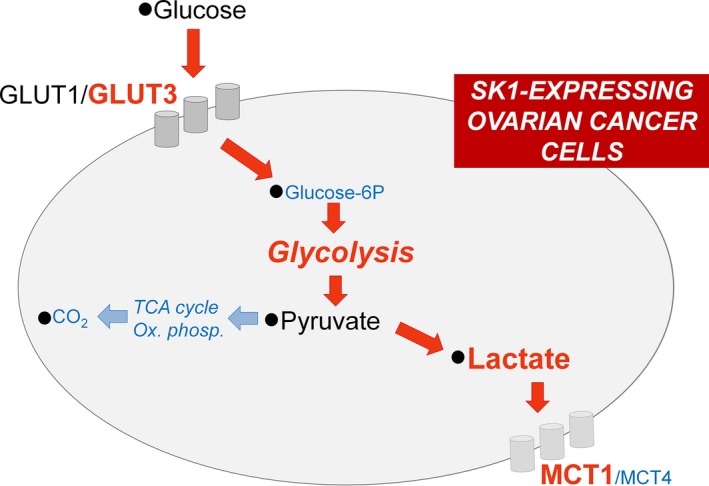
Metabolic shift induced by SK1 expression in A2780 ovarian cancer cells. Red arrows represent augmented fluxes, while cyan arrows indicate diminished processes in consequence of SK1 expression. Metabolite/protein levels and metabolic pathways are shown in red or cyan if they are, respectively, augmented or decreased in SK1‐expressing ovarian cancer cells.

Here, we demonstrate that SK1 plays a crucial role in the metabolic shift toward an aberrant metabolic phenotype driving ovarian cancer cells toward aerobic glycolysis and more sustained macromolecule biosynthesis (Fig. 6). These findings rely on original and innovative combination of untargeted NMR metabolomics and targeted biochemical methodologies. All the observed metabolic features induced by SK1 expression in ovarian cancer cells were found to be independent from the culture conditions (growing or serum starvation), indicating that SK1 is sufficient to induce the specific metabolic phenotype.

In accordance with our findings, it has been recently reported that S1P promotes erythrocyte glycolysis for adaptation to high‐altitude hypoxia (Sun *et al*., 2016), thus providing novel mechanistic insights on how increased glycolytic fluxes might be related to SK1‐mediated generation of S1P. Although this newly discovered mechanism of action of S1P could be restricted to mature erythrocytes as they store highest amounts of S1P, it confirms the relevance of this signaling pathway in the regulation of metabolic processes such as glycolysis.

A suggested role of SK1 in aerobic glycolysis comes from studies on prostate LNCaP cancer cells. Treatment of LNCaP with the SKs pan‐inhibitor SKi affects the levels of few intermediates of the glycolytic pathway and augments the proteasomal activity with consequent degradation of several proteins, including SK1 (Watson *et al*., 2013). However, recent studies have shown a number of important off‐target effects of SKi (Pitman *et al*., 2016). The present study provides the first direct evidence of the role of SK1 in cancer cell metabolism reprogramming. The untargeted metabolic profiling provides a general picture of the complex metabolic network driving to cancer metabolic adaptation and development, which goes beyond the regulation of the glycolysis. The SK1‐induced alteration in a number of metabolic processes is demonstrated by complementary biochemical assays. The high glycolytic rate, with increased levels of lactate along with increased expression of the proton/monocarboxylate symporter MCT1, is paralleled by decreased oxidative metabolism, with accumulation of intermediates of the TCA and reduction in CO_2_ production. Additionally, SK1‐expressing A2780 ovarian cancer cells showed a significant increase in glucose uptake associated with GLUT3 transporter upregulation. SK1 expression caused also a strong induction of S1P_3_ expression as well as a sustained activation of Akt signaling pathway.

The identification of SK1 as a key regulator of cancer metabolic phenotype opens new routes for the development of therapies targeting the metabolic adaptability accompanying the oncogenic process.

## Author contributions

CB and PT conceived and designed the project; CB, VG, and LJ acquired the data; CB, VG, FC, CD, PB, and PT analyzed and interpreted the data; CB, CD, and PT wrote the manuscript. All authors have read and approved the final manuscript.

## Funding

The study was supported by Ente Cassa di Risparmio di Firenze (Grant Numbers 2014.0732 to CD and 2014.0162 to PT); University of Florence (Fondi di Ateneo ex 60%) to PB, FC, and CD. The NMR work was conducted at CERM, Core Centre of the ESFRI Infrastructure Instruct.

## Supporting information


**Fig. S1.** A2780 cells were stably transfected with the empty expression vector (mock) or with pcDNA3‐hSK1^WT^ plasmid (SK1).
**Fig. S2.** Score plot of unsupervised PCA analysis of mock‐ and SK1‐cell lysates: PC1, PC2 and PC3.
**Fig. S3.** Bar plot of ‐Log_2_ (FC) of the analyzed metabolites.
**Fig. S4.** Mock and SK1‐expressing cells were serum‐starved for 24 h before the cells were harvested and then subjected to Sph and Cer analysis.
**Fig. S5.** (A) Bar plots of −Log_2_ (FC) of the metabolites related to glycolysis and to TCA cycle (left and right panel, respectively). Metabolites with −Log_2_ (FC) negative values have lower concentration in SK1 + VPC96091 samples with respect to SK1 samples. Metabolites with −Log_2_ (FC) positive values have higher concentration in SK1 + VPC96091 samples with respect to SK1 samples. Metabolites whose concentration is significantly different (*P*‐value < 0.05, by Paired Wilcoxon test) in SK1 + VPC96091 cells with respect to SK1 cells are marked with *. (B) Box plots: relative concentration levels of the indicated metabolites in each group were calculated by integrating the signal area in the ^1^H NMR spectra. Metabolites whose concentration is significantly different (*P*‐value < 0.05, by Paired Wilcoxon test) in SK1 cells with respect to mock cells are marked with # while metabolites whose concentration is significantly different (*P*‐value < 0.05, by Paired Wilcoxon test) in SK1 + VPC96091 cells with respect to SK1 cells are marked with *.
**Fig. S6.** Box plots: relative concentration levels of the indicated metabolites in each group were calculated by integrating the signal area in the ^1^H NMR spectra.
**Table S1.** Analyzed metabolites.
