# Supplementary material for: NMR metabolomics highlights sphingosine kinase‐1 as a new molecular switch in the orchestration of aberrant metabolic phenotype in cancer cells
Source: Mol Oncol. 2017 Mar 30;11(5):517–33. doi: 10.1002/1878-0261.12048 (PMC5527469; doi:10.1002/1878-0261.12048)
Supplement: Supplementary file 1 — Fig. S1. A2780 cells were stably transfected with the empty expression vector (mock) or with pcDNA3‐hSK1WT plasmid (SK1). Fig. S2. Score plot of unsupervised PCA analysis of mock‐ and SK1‐cell lysates: PC1, PC2 and PC3. Fig. S3. Bar plot of ‐Log2 (FC) of the analyzed metabolites. Fig. S4. Mock and SK1‐expressing cells were serum‐starved for 24 h before the cells were harvested and then subjected to Sph and Cer analysis. Fig. S5. (A) Bar plots of −Log2 (FC) of the metabolites related to glycolysis and to TCA cycle (left and right panel, respectively). Metabolites with −Log2 (FC) negative values have lower concentration in SK1 + VPC96091 samples with respect to SK1 samples. Metabolites with −Log2 (FC) positive values have higher concentration in SK1 + VPC96091 samples with respect to SK1 samples. Metabolites whose concentration is significantly different (P‐value < 0.05, by Paired Wilcoxon test) in SK1 + VPC96091 cells with respect to SK1 cells are marked with *. (B) Box plots: relative concentration levels of the indicated metabolites in each group were calculated by integrating the signal area in the 1H NMR spectra. Metabolites whose concentration is significantly different (P‐value < 0.05, by Paired Wilcoxon test) in SK1 cells with respect to mock cells are marked with # while metabolites whose concentration is significantly different (P‐value < 0.05, by Paired Wilcoxon test) in SK1 + VPC96091 cells with respect to SK1 cells are marked with *. Fig. S6. Box plots: relative concentration levels of the indicated metabolites in each group were calculated by integrating the signal area in the 1H NMR spectra. Table S1. Analyzed metabolites. [file MOL2-11-517-s001.pdf]

SUPPLEMENTARY MATERIAL

NMR Metabolomics Highlights Sphingosine Kinase-1 as a New Molecular Switch in the Orchestration of Aberrant Metabolic Phenotype in Cancer Cells.

Caterina Bernacchioni[1]*, Veronica Ghini[2], Francesca Cencetti[1], Lukasz Japtok[3], Chiara Donati[1], Paola Bruni[1], Paola Turano[2]*.

[1]Department of Biomedical, Clinical and Experimental Sciences, University of Florence, Viale GB Morgagni 50, Florence, 50134, Italy

[2]CERM and Department of Chemistry, University of Florence, Via Luigi Sacconi 6, Sesto Fiorentino, Florence, 50019, Italy

[3]Faculty of Mathematics and Natural Science, Institute of Nutritional Science, Department of Toxicology, University of Potsdam, Arthur-Scheunert Allee 114-116, Nuthetal, Potsdam 14558, Germany

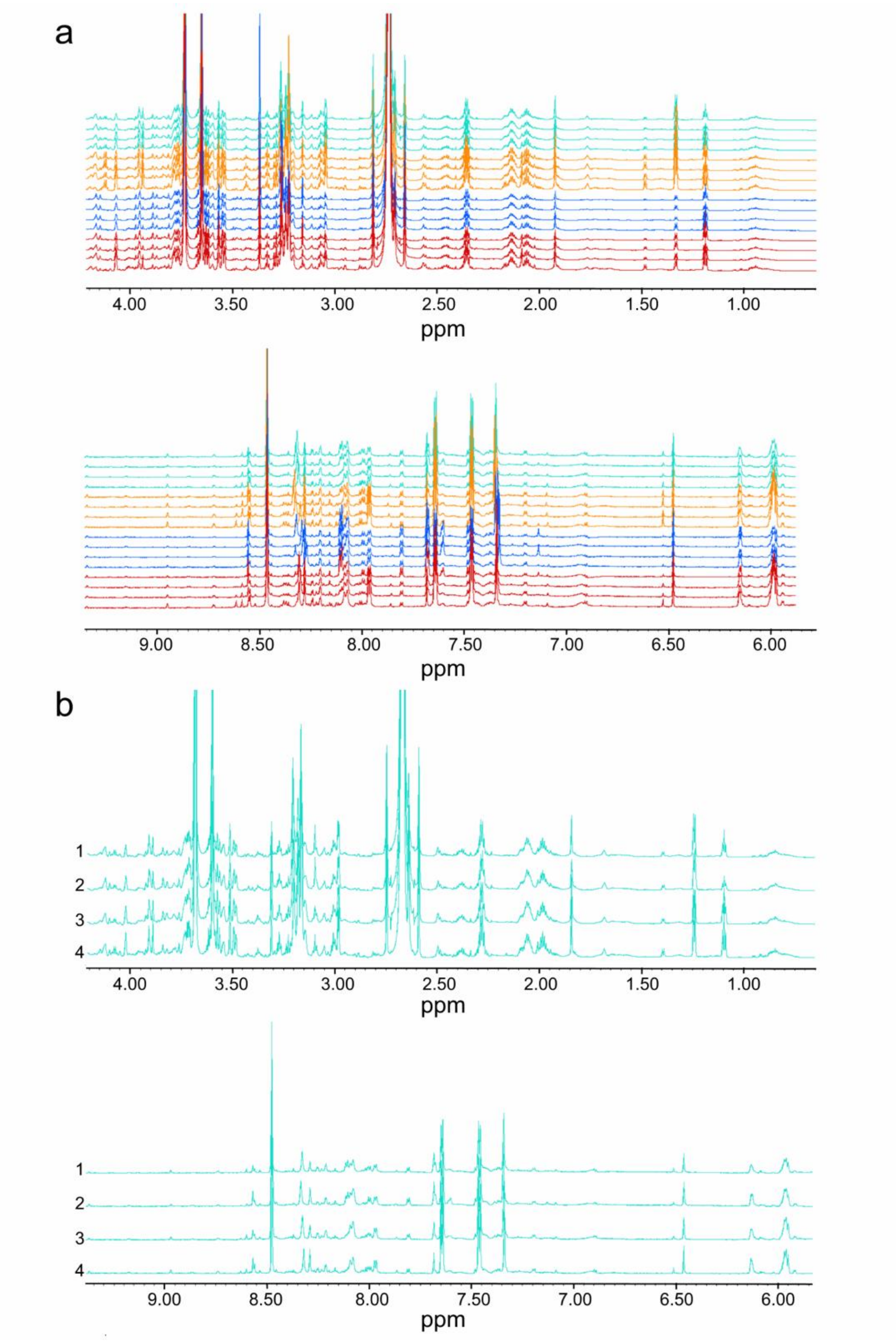

Figure S1. A2780 cells were stably transfected with the empty expression vector (mock) or with pcDNA3-hSK1^{WT} plasmid (SK1). (a) Upfield (1.00-4.00 ppm, upper panel) and downfield (6.00-9.00 ppm, lower panel) regions of the ^{1}H-NMR CPMG spectra; for better peak visualization a 4x vertical expansion is used

for the downfield region. The NMR spectra were acquired in four independently prepared cell lysates for each condition: cyan tracks: mock growing; orange tracks: SK1 growing; blue tracks: mock serum-starved; red SK1 serum-starved. (b). Enlargement of the four ^{1}H-NMR CPMG spectra illustrated in (a), mock growing condition, to show viability and internal high reproducibility.

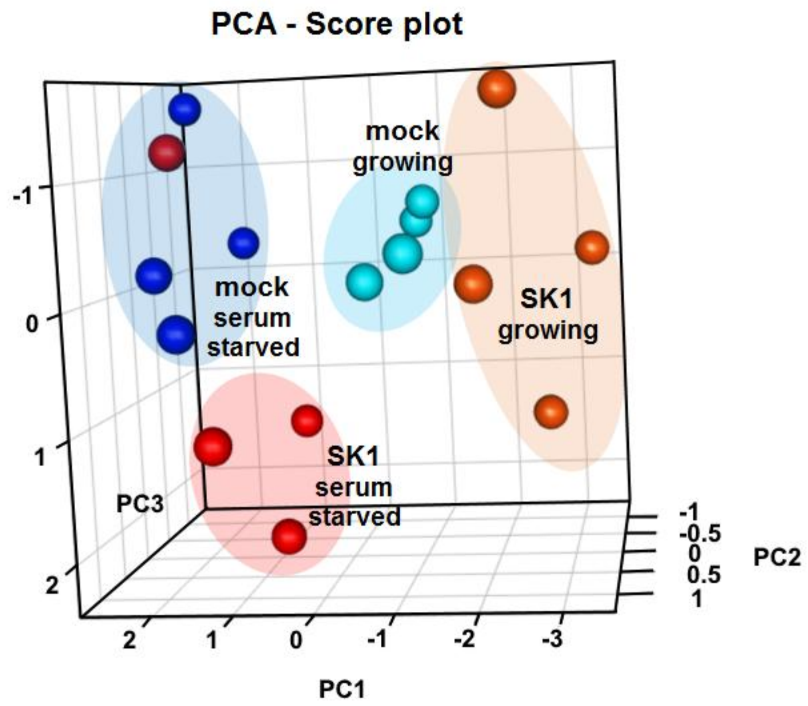

Figure S2. Score plot of unsupervised PCA analysis of mock- and SK1-cell lysates: PC1, PC2 and PC3. Cyan dots, mock group- growing; orange dots, SK1 group- growing; blue dots, mock group- serum starved; red dots, SK1 group- serum starved. The plot has been constructed using ^{1}H NMR CPMG spectra.

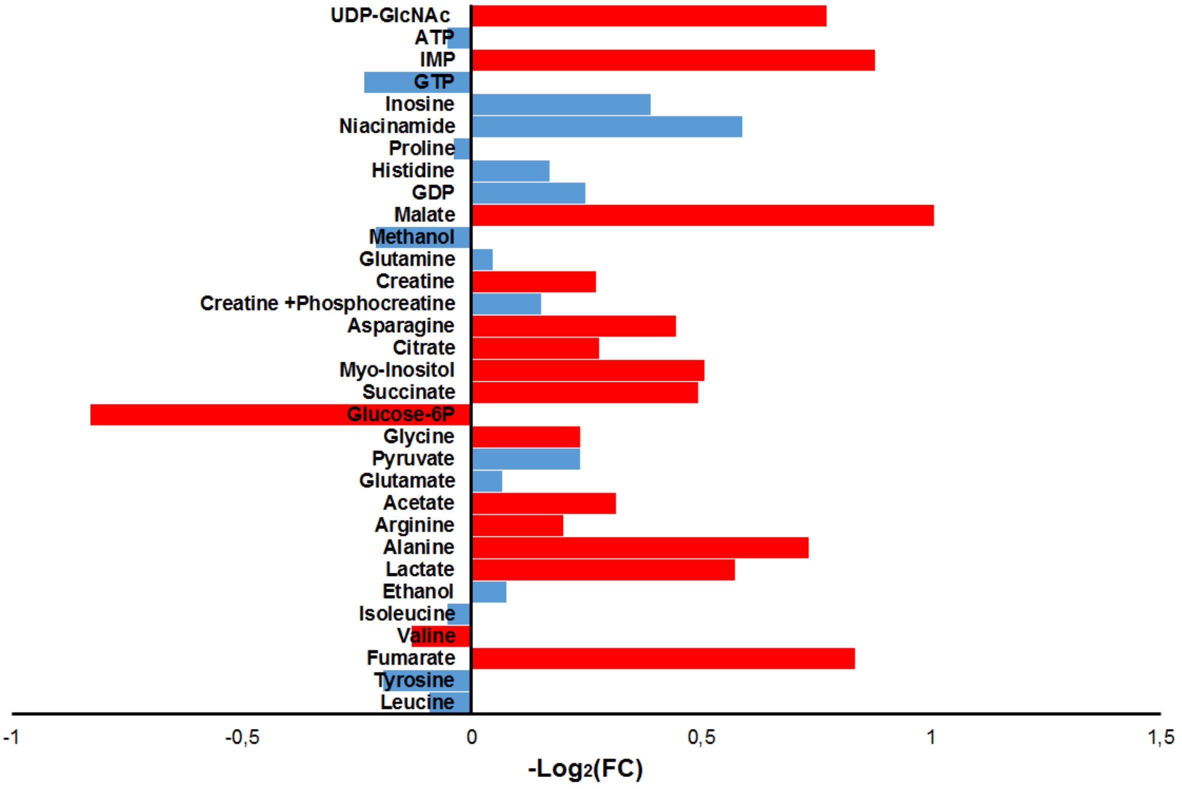

Figure S3. Bar plot of -Log$_2$ (FC) of the analysed metabolites. Metabolites with –Log$_2$ (FC) negative values have lower concentration in SK1 samples with respect to mock samples. Metabolites with –Log$_2$ (FC) positive values have higher concentration in SK1 samples with respect to mock samples. Red bars represent metabolites whose concentration is significantly different (p-value < 0.05) in SK1-expressing cell lysates with respect to mock cell. Metabolites whose concentration is not significantly different (p-value > 0.05) in the two groups of samples are shown in blue.

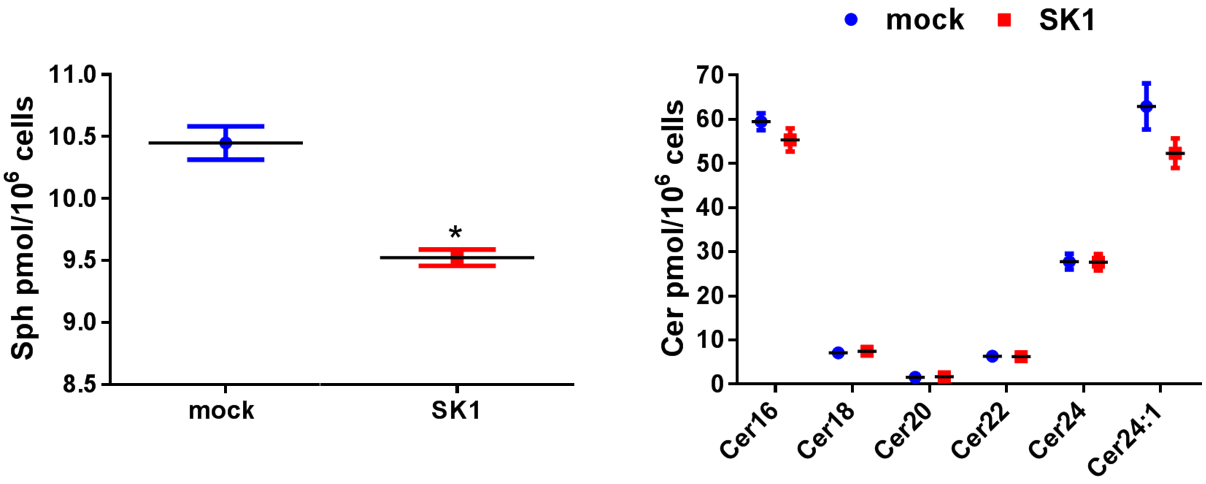

Figure S4. Mock and SK1-expressing cells were serum-starved for 24 h before the cells were harvested and then subjected to Sph and Cer analysis. The effect of SK1 overexpression on Sph levels inside the cells was statistically significant by Student's *t* test *p < 0.05.

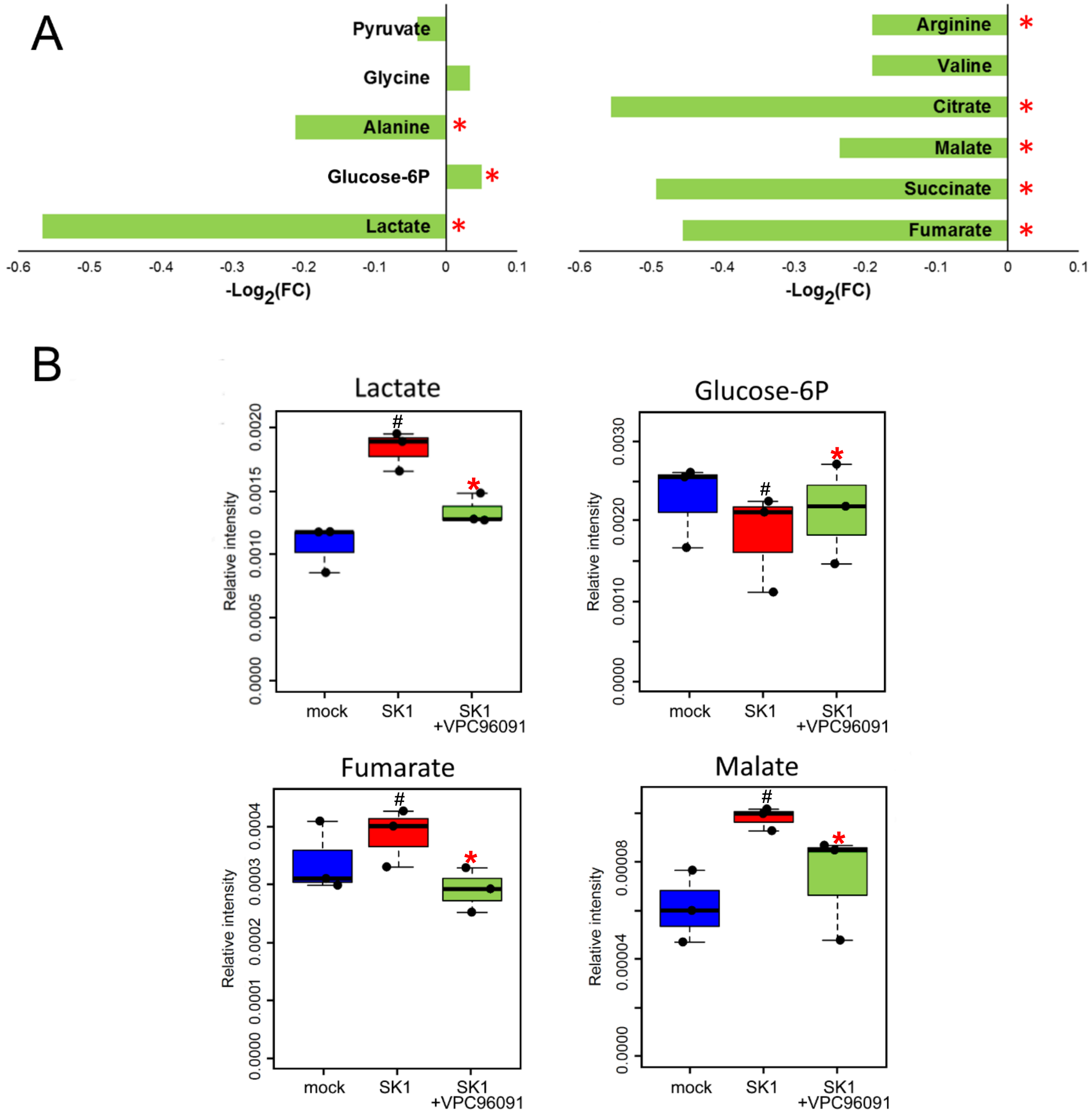

Figure S5. (A) Bar plots of -Log_2 (FC) of the metabolites related to glycolysis and to TCA cycle (left and right panel, respectively). Metabolites with –Log_2 (FC) negative values have lower concentration in SK1+VPC96091 samples with respect to SK1 samples. Metabolites with –Log_2 (FC) positive values have higher concentration in SK1+VPC96091 samples with respect to SK1 samples. Metabolites whose concentration is significantly different (p-value < 0.05, by Paired Wilcoxon test) in SK1+VPC96091 cells with respect to SK1 cells are marked with *. (B) Box plots: relative concentration levels of the indicated metabolites in each group were calculated by integrating the signal area in the ^{1}H NMR spectra.

Metabolites whose concentration is significantly different (p-value < 0.05, by Paired Wilcoxon test) in SK1 cells with respect to mock cells are marked with # while metabolites whose concentration is significantly different (p-value < 0.05, by Paired Wilcoxon test) in SK1+VPC96091 cells with respect to SK1 cells are marked with *.

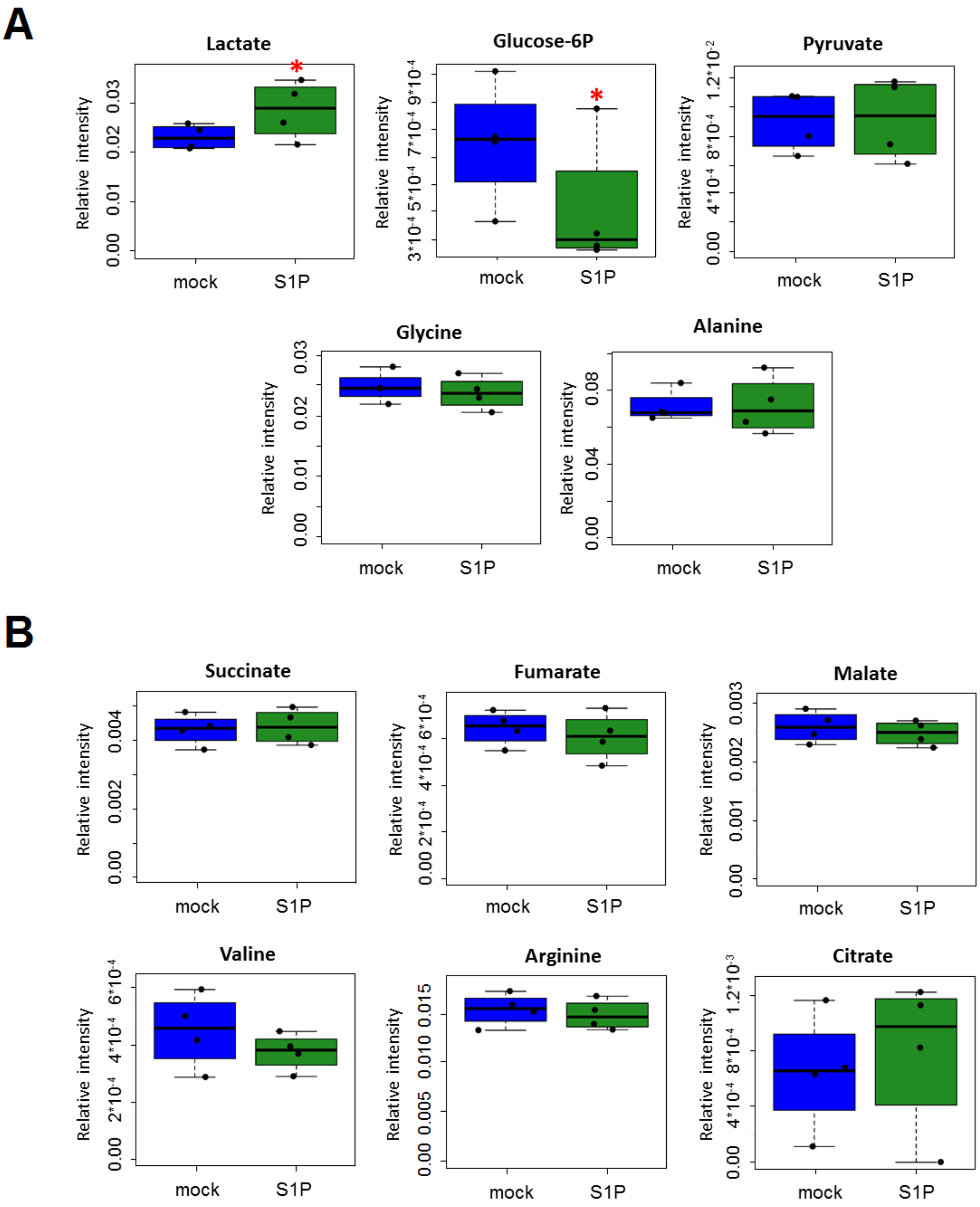

Figure S6. Box plots: relative concentration levels of the indicated metabolites in each group were calculated by integrating the signal area in the ^{1}H NMR spectra. (A) Metabolites related to glycolysis and (B) metabolites related to TCA cycle. Changes in metabolite levels caused by the addition of S1P were statistically significant by Paired Wilcoxon test, *$p<0.05$; lactate p= 0.03; glucose-6P p= 0.04.

Metabolite	Database	Compound ID
**Acetate (Acetic acid)**	HMDB	HMDB00042
**L-Alanine**	HMDB	HMDB00161
**L-Arginine**	HMDB	HMDB00517
**L-Asparagine**	HMDB	HMDB00168
**ATP (Adenosine triphosphate)**	HMDB	HMDB00538
**Citrate (Citric acid)**	HMDB	HMDB00094
**Creatine**	HMDB	HMDB00064
**Phosphocreatine**	HMDB	HMDB01511
**Ethanol**	HMDB	HMDB00108
**Fumarate (Fumaric acid)**	HMDB	HMDB00134
**GDP (Guanosine diphosphate)**	HMDB	HMDB01201
**Glucose-6 phosphate**	HMDB	HMDB01401
**Glutamate (D-Glutamic acid)**	HMDB	HMDB03339
**L-Glutamine**	HMDB	HMDB00641
**Glycine**	HMDB	HMDB00123
**GTP (Guanosine triphosphate)**	HMDB	HMDB01273
**L-Histidine**	HMDB	HMDB00177
**IMP (Inosinic acid)**	HMDB	HMDB00175
**Inosine**	HMDB	HMDB00195
**L-Isoleucine**	HMDB	HMDB00172
**Lactate (Lactic acid)**	HMDB	HMDB00190
**L-Leucine**	HMDB	HMDB00687
**Malate (D-Malic acid)**	HMDB	HMDB31518
**Methanol**	HMDB	HMDB01875
**Myo-inositol**	HMDB	HMDB00211
**Niacinamide**	HMDB	HMDB01406

**L-Proline**	HMDB	HMDB00162
**Pyruvate (Pyruvic acid)**	HMDB	HMDB00243
**Succinate (Succinic acid)**	HMDB	HMDB00254
**L-Tyrosine**	HMDB	HMDB00158
**UDP-GlcNAc (Uridine diphosphate-N-acetylglucosamine)**	HMDB	HMDB00290
**L-Valine**	HMDB	HMDB00883

Table S1. Analysed Metabolites. The signal of these metabolites in the CPMG spectra were well defined and resolved; their relative levels in the different lysate groups were analysed. The Human Metabolome Database (HMDB) compound ID of each metabolite is also reported.
